# Adversarial Data Hiding in Digital Images

**DOI:** 10.3390/e24060749

**Published:** 2022-05-25

**Authors:** Dan Wang, Ming Li, Yushu Zhang

**Affiliations:** 1 College of Software, Henan Normal University, Xinxiang 453007, China; wangdan@asyncx.top; 2 College of Computer and Information Engineering, Henan Normal University, Xinxiang 453007, China; 3 Key Laboratory of Artificial Intelligence and Personalized Learning in Education of Henan Province, Xinxiang 453007, China; 4 College of Computer Science and Technology, Nanjing University of Aeronautics and Astronautics, Nanjing 211106, China; yushuboshi@163.com

**Keywords:** adversarial example, convolutional neural network, data hiding, LSB

## Abstract

In recent studies of generative adversarial networks (GAN), researchers have attempted to combine adversarial perturbation with data hiding in order to protect the privacy and authenticity of the host image simultaneously. However, most of the studied approaches can only achieve adversarial perturbation through a visible watermark; the quality of the host image is low, and the concealment of data hiding cannot be achieved. In this work, we propose a true data hiding method with adversarial effect for generating high-quality covers. Based on GAN, the data hiding area is selected precisely by limiting the modification strength in order to preserve the fidelity of the image. We devise a genetic algorithm that can explore decision boundaries in an artificially constrained search space to improve the attack effect as well as construct aggressive covert adversarial samples by detecting “sensitive pixels” in ordinary samples to place discontinuous perturbations. The results reveal that the stego-image has good visual quality and attack effect. To the best of our knowledge, this is the first attempt to use covert data hiding to generate adversarial samples based on GAN.

## 1. Introduction

With the advancement of information technology, the issue of digital media privacy and security is becoming increasingly important. According to studies, social media sites distribute over 100 million photos every hour and over 3 billion photos per day. To compensate for the limited storage space on mobile devices, a large number of photographs concerning personal privacy are transmitted and shared in the cloud via third-party storage (service) providers, with automated synchronization possible. However, in recent years data leakage has posed a severe threat to cloud server users, and personal privacy is facing an unprecedented crisis. Despite the efforts of third-party storage (service) providers to address the issue of user privacy breaches, users’ personal information remains available to individuals with ulterior purposes. They have access to all of a user’s images, including private photos, which can lead to serious consequences. In particular, recent advances in pattern recognition technology based on deep learning can be used by attackers for purposeful target recognition and feature extraction from massive network pictures.

Adversarial samples have recently been proven to preserve users’ personal privacy and to effectively prevent unscrupulous parties from performing illicit target recognition on image big data using neural networks [1]. This function is achieved by introducing adversarial noise to ordinary samples [2], causing CNNs to misclassify images and hence impede automatic face recognition [3], protecting the privacy of photos on social networks [4]. The user cannot notice the effect of the adversarial disturbance [5]. This characteristic allows adversarial perturbation to strike a fair balance between privacy and image usability.

Adversarial perturbation adds a certain amount of noise signal to the host image. Similarly, the technique of data hiding adds extra information to the host image, as well as providing image integrity, copyright protection, and other benefits. As a result, the combination of information concealing and adversarial samples is favored. Researchers have made useful attempts to combine adversarial samples with generative data hiding, which is based on neural networks. Ian GoodFellow first proposed Generative Adversarial Nets (GANs) [6] in 2014. GANs have subsequently become a research hotspot, and are widely used in the field of multimedia security. Several researchers have aimed to explore the similarities and differences between data hiding and machine learning. Quiring et al. [7] examined the parallels and contrasts between watermarking and machine learning attacks in their research. Although the two attacks have distinct goals, the potential attack techniques are remarkably similar in that they both modify the target item in order to cross the decision boundary for the least cost. This has provided academics with the hope of combining data hiding with machine learning. In 2017, Abadi M [8] was the first to propose the use of GAN in information security, developing a neural network-based encryption model. By fighting against a generator and a discriminator in a messaging game, he demonstrated that the neural network could learn how to encrypt. Steganographic Generative Adversarial Networks (SGAN) [9] introduced deep learning to the field of generative data hiding in the same year. Based on Deep Convolutional Generative Adversarial Networks (DCGAN) [10], Volkhonskiy designed a discriminator network implemented via CNN as an extra discriminator to perform steganalysis. The generated images were appropriate for data hiding. Based on SGAN, Shi et al. [11] proposed Secure Steganography based on Generative Adversarial Networks (SSGAN). SSGAN replaces DCGAN with Wasserstein GAN (WGAN) [12] and adds GNCNN [13] as a new discriminator. While the SGAN and SSGAN algorithms demonstrate the potential of generative adversarial networks in the field of information hiding, they both utilise standard steganographic procedures. Tang et al. [14] introduced the ASDL–GAN model, which uses GAN paired with the standard carrier-free information hiding approach STC algorithm to automatically learn steganography. Hayes et al. [15] presented Ste-GAN-ography in 2017, which is a radical departure from typical generative information hiding techniques. This model is a generator–discriminator adversarial network based on generative adversarial networks. HiDDeN, an information hiding technique with the same loss function as Ste-GAN-ography and a different model structure, was proposed by Zhu et al. [16] in 2018. Baluja’s [17] and Wu’s [18] approaches both focus solely on embedding secret images into cover images. Their tests revealed that images have considerable spatial correlation, and they used this characteristic to train convolutional neural networks to hide secret images in carrier images.

Although the above initial attempts to combine adversarial perturbation with information hiding have made significant progress, they all hide information based on neural networks without carriers, in which there is neither subjective control nor protection of the integrity and copyright of carrier data. In other words, information hiding only achieves covert communication in this case; the second key purpose of information hiding, carrier protection, is ignored.

If the carrier data are to be protected, we must make the information operate while hidden in the substantial target carrier. It is worth noting that subsequent research [19] has found that adversarial samples are kept after various picture manipulations, which provides a foundation for the simultaneously hidden information with adversarial samples proposed here. In addition, Zhu et al. [20] have argued that while the existence of adversarial samples in a neural network is considered a disadvantage, it may not be one in the field of information hiding. If neural networks can be tricked by small disturbances into classifying correctly, this could be applied in information hiding to trick a neural network-based steganography analyzer into incorrect classification. In other words, information hiding can be used as a small perturbation in the substance carrier.

In recent years, several studies have emerged on achieving adversariality through information hiding in substantial carriers. Many researchers have realized that current adversarial perturbations usually take the form of noise that has no practical meaning. Image watermarking is a common copyright-protection approach. If the image watermark is considered as meaningful noise added to the original samples, the final image will not impact understanding of the image’s content or arouse suspicion. Jia et al. [21] presented basin-hopping evolution (BHE), a novel optimization algorithm that combines explicit image watermarking techniques and adversarial sample algorithms, and applied a watermark perturbation (Adv-watermark) for adversarial samples based on BHE. Jiang et al. [22] introduced a fast adversarial watermark attack (FAWA) method based on the fast differential evolution technique, which fools CNNs by layering watermarks on images. This approach of fooling VGG-16 had a success rate of 41.3%, which indicates good portability. It has been shown that most studies can only use a mix of explicit watermarking and deep learning techniques to increase aggression by increasing the watermark’s strength in order to solve the problem of information hiding adversarial effect. It is obvious that the previous techniques have a greater negative impact on image quality when combined. Even if the embedded information has no effect on the use of the original digital image, it obstructs visual appreciation and decreases the image’s viewability. As a result, explicit watermarking throws off the balance of privacy, security, and image quality. It seems impossible to guarantee both. If the added disturbance is considerable, the image quality will be reduced. However, if there is no clear and explicit watermark, security is not guaranteed at all. Explicit watermarking, on the other hand, has a serious flaw. “Model extraction” and “watermark estimation” can be used to fight against network attacks and steganalysis, respectively [7]. They can steal the model or remove the watermark in both circumstances by learning to generate a near-perfect “forgery”. As a result, explicit watermarking reduces security significantly, whereas implicit watermarking does not.

In this paper, we propose an algorithm with carrier information hiding which can hide the data (implicit watermark) and at the same time turn ordinary samples into adversarial samples with high image quality. In addition, the applicable convolutional neural network is wider. This is a true combination of information hiding and adversarial samples, resulting in greatly increased flexibility, security, information hiding, and image quality. The adversarial stego-images obtained using this adversarial data hiding (ADH) technique have good visual quality when compared to normal ways of generating adversarial samples, and the additional perturbations are nearly invisible. There is a certain opposition between the quality of the adversarial sample image and the attack ability; the algorithm can generate perturbations that successfully fool the discriminator, the perturbations can make the steganography synthesize a high quality adversarial stego-image, and the hidden information can be obtained by decoding. We chose to search in 3D space in order to establish better decision boundaries; that is, three “sensitive pixels” are selected based on the differential genetic algorithm’s properties. The training network section uses the pre-existing network, and the focus of this paper is on how to generate the adversarial sample without damaging the image quality while hiding the information. The content is split into two sections, namely, generating adversarial samples and embedding information, which can be used to confine modified units and reduce the perturbation, respectively. We propose a new genetic algorithm, the boundary position differential evolution algorithm, to locate the decision boundary affecting the deep neural network judgment in the search space, find the three most sensitive discontinuous pixel points on the decision boundary, and then embed the information using the Least Significant Bit (LSB) technique. This approach ensures the security of the secret information and the concealment of the adversarial sample. In this way, we use only probability labels to generate adversarial samples. We used ADH for the images in the CIFAR-10 dataset in the validation experiments section, which comprises 60,000 32 × 32 color images divided into ten classes of 6000 images each. Using this training set increases the possibility that the discriminator has been sufficiently trained, and makes subsequent modification operations on multiple non-contiguous pixels easier. Several experimental results show that the proposed ADH produces satisfying performance and exceeds previously existing methods.

The rest of this paper is organized as follows. The proposed method is introduced in detail in Section 2. In Section 3, the experimental results and analysis are provided. Finally, we conclude the paper in Section 4.

## 2. Proposed Method

**Figure 1:**
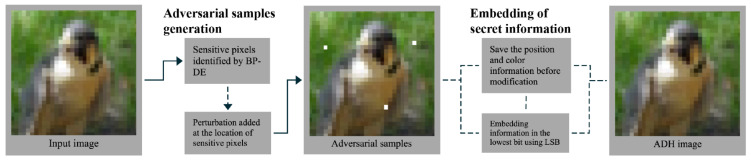
Flowchart of ADH.

In this section, the proposed ADH approach is described. As in Figure 1, we first constrain the effective embedding region with adversarial effect in the host image using a new BP-DE algorithm, then embed the additional data into the selected region using the LSB algorithm.

Our proposed adversarial information hiding is essentially based on the LSB information hiding method for achieving adversariality. As the LSB algorithm is a basic method of information hiding, we focus on describing how to find the appropriate embedding location and do not describe extensively how to achieve information hiding.

### 2.1. Constraining the Image Embedding Area

Specific perturbation can cause a classification model to misclassify a newly constructed sample. The training model’s random selection cannot actually cover all possibilities, and most likely only a fraction of them. Deep neural networks are vulnerable to adversarial sample attacks because there is a gap between the boundaries of the trained model and the boundaries of the real decision model. We used the optimization method to generate adversarial samples. The principle is to continually generate adversarial samples after perturbing pixels on ordinary samples, then compare the results of each adversarial sample to determine the optimal small pixel modification value.

ADH is a semi-black-box perturbation, which means that the operator must not know the entire target CNN and has no right to query its structure or gradient. ADH generates adversarial samples relying only on the probability labels, meaning that it can be applied to more settings and can be applied to CNNs for which the details are not known. There is no need to measure the fitness, because it does not abstract the problem of searching for the optimum position into any calculation about fitness. Instead, whether the probability label of this adversarial sample changes or not is regarded as the basis of success.

Usually, the adversarial samples are constrained to perturb all pixels by cumulatively modifying the intensity of the pixel points together.

We classify this problem as a single-objective constrained optimization problem, as there is have one objective function, namely, the result of the interference image discriminator; that is, we continue to confine the search space in order to find the best value for the unique goal function.

The generation of adversarial samples can be regarded as an optimization problem with the following constraints:

(1) $$\begin{array}{c} \arg\min_{t}d\left(t\right)\\ s.t.\;f_{\mathrm{adv}\;}\left(x+t\right)=y^{*} \end{array}$$

where $f_{\mathrm{adv}\;}$ is the image discriminator that accepts the input and d(t) is the perturbation vector, *t*, that must be applied to the original sample, *x*, in order to achieve the incorrect prediction, $y^{*}$; $y^{*}$ can be any classification except for the proper one, as this is a non-targeted attack.

As stated in Section 1, we want to guarantee the image quality; thus, the optimal choice is to limit the modifications to be sufficiently tiny, and the intensity cannot be as high as in the previous methods. The first problem to address is ensuring that the constraint range is small enough. We measured perturbation strength using the number of pixels instead of the length of the perturbation vector, which is the traditional method, as this makes subsequent information hiding easier. As not every feature change affects the classifier’s output, the discriminator will only be mistaken if the selected pixel surpasses the decision border [23].

The first step in improving picture quality is to change the constraint unit to a size that makes the image disturbance nearly undetectable under visual inspection by changing a few pixels on the image.

However, this reduces the aggressiveness of the adversarial sample; thus, we used constraint optimization to preserve the aggressiveness by searching the decision boundary and selecting a specific point after adjusting its feature values. The major difference between constraint optimization class problems and unconstrained problems is that the optimal solution must satisfy all constraints. A constrained optimization problem’s constraints divide the space of decision variable values into feasible and infeasible domains, with the purpose of the solution being to identify the decision value that makes the goal optimal in the feasible domain. Constraints not only make the feasible domain smaller and the search space more complex, but they make the modified areas difficult to handle, making the problem much more difficult to solve and limiting the effectiveness of the optimization process in finding the global optimal solution. Obviously, the simultaneous consideration of feasibility and optimality is the major reason why constrained optimization issues are more difficult to solve [24].

Intelligent optimization algorithms represent an excellent choice for breaking the traditional constraints of optimization algorithms and increasing efficiency. We decided to use a genetic algorithm to solve the problem of finding the decision boundaries. This allows for finding the decision limits of natural images in a single optimization, which is addressed in detail in Section 2.2.

The following is the generating formula for this paper’s adversarial sample:

(2) $$\begin{array}{c} \arg\min_{t}d\left(t\right)\\ s.t.\;f_{\mathrm{adv}\;}\left(x+t\right)=y^{*}\\ t\leq N \end{array}$$

**Figure 2:**
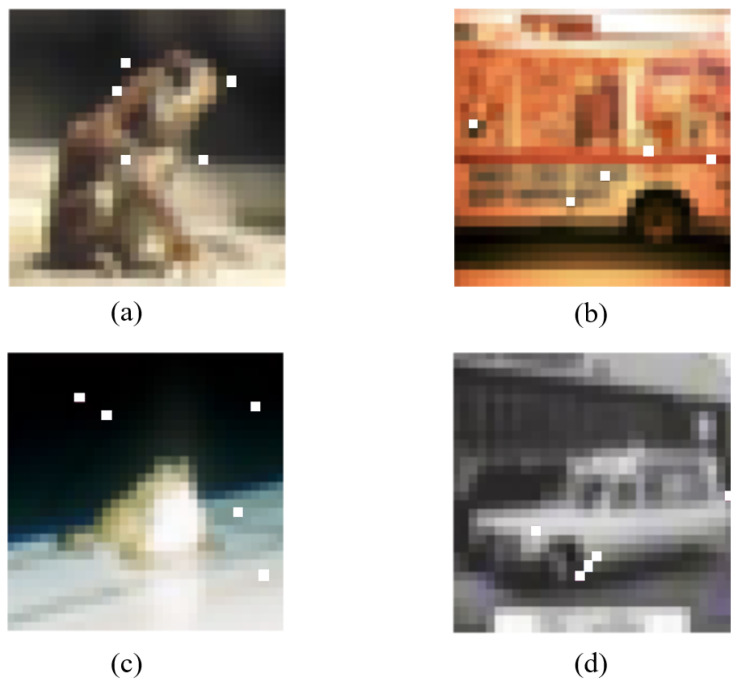
Decision boundaries (five pixels): (**a**) The true label is frog, the predicted label is deer; (**b**) The true label is truck, the predicted label is airplane; (**c**) The true label is frog, the predicted label is dog; (**d**) The true label is automobile, the predicted label is truck.

**Figure 3:**
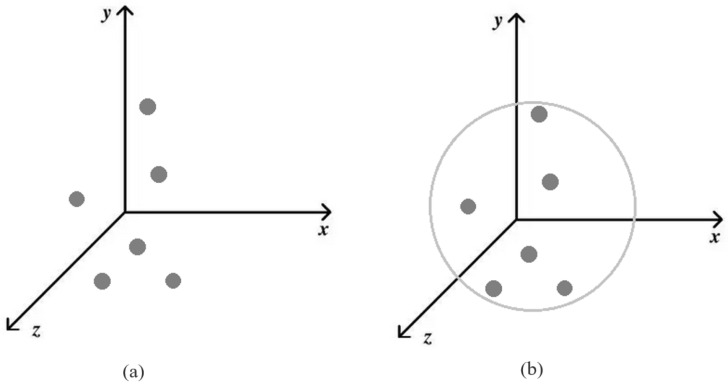
Three-dimensional single-objective decision space and target space: (**a**) three-dimensional single-objective decision space (the gray dots represent “sensitive pixels”); (**b**) three-dimensional single-objective target space (the gray circle represents the constraint).

For the original sample *x*, d(t) represents the required number of perturbed pixels, *t*, and *N* is the minimum number of pixels that have been modified. Previous work changed a fraction of all dimensions; however, the current method only changes dimension *t*, maintaining the other dimensions of *x* unchanged. As in Figure 2, these images show what a modified dimension would look like if it were in five dimensions. Because we decided to create a three-dimensional search space, in this paper t=3. As in Figure 3, the three-dimensional Euclidean space is a three-dimensional space. When there are only sensitive pixels and no limit, as shown in Figure 3a, it is difficult to find them or the image quality is greatly damaged when they are changed. Limiting them to a suitable range, as shown in Figure 3b, allows for minor modifications.

Both figures depict the search space as a three-dimensional space. The “sensitive pixels” in the first figure are not evenly distributed in three-dimensional space. The second figure describes a spherical area that is constrained to include all of them, making searching easier. The position of a point in Euclidean space is determined by three coordinates. Three-dimensional space, with its three dimensions, length, breadth, and height, is objectively existing real space. We can have a linguistic explanation of “sensitive pixels” when the search space is three-dimensional, and the search space is slightly higher than the dimension of the image in order to ensure more precise findings.

Additionally, both generative adversarial networks and data hiding perform better, and decision boundaries can be discovered faster. The next section describes the specific algorithm used for decision boundary localization.

### 2.2. BP–DE Algorithm

Boundary position differential evolution (BP–DE) is a genetic algorithm that searches for decision boundaries using differential evolution (DE). The DE  [25] algorithm is a population differences-based heuristic method. It is a stochastic model that models biological evolution and works in the same way as other evolutionary algorithms. Because of its excellent robustness and ability to identify global optimality, the use of real number encoding means that this algorithm is straightforward to use and requires few regulated parameters. It iterates until it finds the best solution, preserving those members who have adapted to their surroundings. This algorithm is extremely efficient and has a much shorter runtime. DE preserves the global population-based search strategy, the simple difference-based variation operation, and the one-to-one competitive survival strategy, which decreases the complexity of genetic operations when compared to other evolutionary algorithms.

The BP–DE algorithm begins with initialization. The perturbations are encoded in a one-dimensional array that is optimized using differential evolution. The position (x,y coordinates) of a perturbation being added is represented by a one-dimensional array, and a perturbation affects one pixel; thus,

(3) $$X=(x_{ij}^{g},y_{ij}^{g})$$

where the top left corner of the x,y coordinate *g* represents is the *g*th generation, the bottom left corner of *i* represents is the *i*th perturbation, and *j* represents is the *j*th dimension. In general, the user chooses several points to represent the search space for several dimensions. During initialization, the number of perturbations, is set to *n*, then the array remains one-dimensional in order to facilitate algorithmic processing. Then, the array becomes

(4) $$X=\left(x_{11}^{0},y_{11}^{0},x_{22}^{0},y_{22}^{0},\ldots ,x_{nn}^{0},y_{nn}^{0}\right)$$

Assume that the ordinary sample size is L×W pixels. At this time, the range of x,y coordinate values is 0∼L and 0∼W. The number of perturbations is determined by the demand.

After the location of a pixel point has been determined, a perturbation is randomly added to it. Nevertheless, after the form and appearance of this perturbation has been decided, the BP–DE algorithm will continue to follow it and add it to all candidate solutions. The variables are fixed to allow the decision boundary to be found more precisely. Then, the process of generating random initial populations (basis vectors) in the range is

(5) $$x_{ij}^{0}=x_{\min,j}+{\mathrm{rand}}_{ij}\left(0,1\right)\left(x_{\max,j}-x_{\min,j}\right),$$

where 0 denotes the initial population and $x_{\max,j}$ and $x_{\min,j}$ denote the upper and lower constraints for determining the *j*th dimension for individual $x_{i}$. The BP–DE algorithm’s difference characteristic is shown in the difference vector $\left(x_{\max,j}-x_{\min,j}\right)$; ${\mathrm{rand}}_{ij}\left(0,1\right)$ represents taking random values within [0,1]. In general, the DE generates 400 possible solutions as the initial population.

The experimental vector is then generated by selecting the current individual to be updated and cross-recombining it with the mutant vector. When the recombination threshold is exceeded, a random probability value is generated and the mutant vector is assigned to the experiment vector. Otherwise, the experimental vector is allocated to the current individual. The core of this experiment is thus to generate mutant individuals, which might be either the current basis vector or the current individual. At least one dimension of the experimental vector should be retained from the mutant vector by BP–DE. Instead of staying in place, the optimal solution might be achieved slowly via this method. The formula for producing mutant individuals $v_{i}^{g}$ is

(6) $$v_{ij}^{g}=x_{r1,j}^{g}+F\left(x_{r2,j}^{g}-x_{r3,j}^{g}\right)$$

Three other individuals, $r_{1}$, $r_{2}$, and $r_{3}$, in the same population (parent) who are distinct from the target individual are randomly selected in the population. The stride element is denoted by *F*, and the stride factor’s meaning is as follows: In the classic DE, *F* is selected to be isotropic. That means different individuals have different dimensions, whereas the same individual is different while having the same dimensions. This makes it easier for BP–DE to discover the best solution in the same search space. In most cases, the DE generates the same number of candidate solutions (offspring) in each iteration as the initial candidate solutions.

Crossover behavior is not apparent in BP–DE.

Following the generation of mutant individuals, BP–DE forces the offspring to compete with their parents for the quality of the next generation. Only winners survive to the next generation. The sign of competitive victory is that the generated adversarial sample has better attack power in the generative adversarial network and can produce labeling probability with greater error. The selection operation can then be visualized as follows:

(7) $$x_{i}^{g+1}=\left\{\begin{array}{cc} u_{i}^{g} & \;\mathrm{if}\;f\left(u_{i}^{g}\right)⩾f\left(x_{i}^{g}\right)\\ x_{i}^{g} & \;\mathrm{otherwise}\; \end{array}\right.$$

where f(x) is an individual’s fitness function in this population; here, we use it to denote the confidence level of the adversarial sample’s non-target attack.

When the number of algorithm iterations reaches the set maximum or when the probability label of a non-target attack of a randomly chosen ordinary sample in the data set exceeds 50%, the algorithm quits. If the termination condition is not met, the above variation cross-selection operation is repeated until the condition is met.

The following are the key benefits of using BP–DE to generate adversarial images:

1. Simplicity: the approach does not abstract the objective function into a specific equation, and the starting value setting is not difficult. There is no requirement for prior knowledge, and the fitness simply has to know the probability labels created by the unknown CNNs. This is the basis for the ADH algorithm’s ability to perform in a wide range of CNNs as well.
2. Global: The behavior of mutants in the algorithm might avoid falling into the local mechanisms by converging as much as possible in the direction of the global mechanism, giving the algorithm the potential to seek global merit.
3. Fast: Although the algorithm contains randomness, the variation and selection cause the process to accelerate toward the extreme value point, allowing the algorithm to change quickly while tracking the change.

### 2.3. ADH Process

As mentioned above, we constrained the units of modification and used the BP–DE method to search the decision boundary in 3D space, generating an adversarial sample at a cost of three pixel points. In order to reduce the intensity of the modified pixels such that the perturbation is not visible, we used the LSB algorithm for data hiding. The LSB algorithm embeds the information in *N* individual pixel points, which are the ”sensitive pixels” at the decision boundaries just selected by the BP–DE algorithm. The secret information is encoded in the lowest valid bit of each of *N* individual pixels, and changing this position has no influence on the carrier image’s quality. It is only necessary to read the position of these *N* individual pixel points out of storage and then decode them when the data is to be read out. This measure reduces the intensity of modification, improving the security and concealment of ADH. The ADH algorithm, including the information hiding process, is shown in Algorithm A1 of the Appendix A.

## 3. Experimental Results and Analysis

We conducted various experiments for the generated image part and the information embedding part, respectively, in order to evaluate the performance of the proposed ADH. We examined both the stealthiness of the adversarial stego-images and the aggression of the adversarial samples after they had been embedded with data. We examined and analyzed the secret hidden samples using two picture quality assessment methods, described in the concealment test section (Section 3.1). The aggressiveness of the adversarial samples after altering the “sensitive pixels” and the aggressiveness of the covert adversarial samples created by ADH were then examined, as described in the aggressiveness test section (Section 3.2). Finally, we compared the results of this experiment to those of earlier methods in order to illustrate the efficacy of ADH.

For this evaluation, we used the Cifar-10 dataset. The task of the dataset is to correctly classify a 32 × 32 pixel image in 1 of 10 categories (e.g., bird, deer, truck) The CIFAR-10 dataset consists of labeled subsets of the 80 million tiny images, which were collected by Alex Krizhevsky, Vinod Nair, and Geoffrey Hinton [26]. To run the experiment in the tutorial locally, a dedicated GPU suitable for running with Keras (tensorflow-gpu) is recommended and Python 3.5+ is required. As the target image classifier, we trained three types of common networks: LeNet, Net in Net, and Deep Residual Network (ResNet). To achieve improved classification accuracy, the network settings were preserved as close to the original configuration as feasible with minimal changes. For the attack, 500 natural photos were chosen at random from the cifar-10 test dataset. To make the results more visible, we set all of the perturbations added with BP–DE as white pixel points.

### 3.1. Concealment Test

The steganography assessment of the image generated by ADH is simply an image quality evaluation of the hidden adversarial image to see whether the image quality distortion generated during the production process affects the observer’s information acquisition and subjective perception. In terms of approaches, it can be separated into subjective picture quality assessment methods and objective image quality evaluation methods. The subjective image quality assessment method assesses a picture based on how the viewer perceives it. The Peak Signal-to-Noise Ratio (PSNR) and Structural Similarity (SSIM) [27] utilized in this paper are both full-reference based image quality evaluations. PSNR evaluates samples using the original image as a reference, while SSIM is a comparison measurement based on three factors: brightness, contrast, and structure.

With a clean image *I* of size m×n and a noisy image *K*, the Mean Square Error (MSE) is defined as the formula

(8) $$MSE=\frac{1}{mn}\sum_{i=0}^{m-1}\sum_{j=0}^{n-1}{\left[I\left(i,j\right)-K\left(i,j\right)\right]}^{2}$$

Then, the PSNR (dB) is defined as

(9) $$PSNR=10\cdot \log_{10}\left(\frac{MAX_{I}^{2}}{MSE}\right)=20\cdot \log_{10}\left(\frac{MAX_{I}}{\sqrt{MSE}}\right)$$

where $MAX_{I}$ denotes the maximum value of image color and 8-bit sampling points are expressed as 255. Thus, the larger the PSNR value, the less distortion it represents.

Then, the defining equations of the three factors of SSIM are

(10) $$\begin{array}{cc} l(x,y) & =\frac{2\mu_{x}\mu_{y}+c_{1}}{\mu_{x}^{2}+\mu_{y}^{2}+c_{1}} \end{array}$$

(11) $$\begin{array}{cc} c(x,y) & =\frac{2\sigma_{x}\sigma_{y}+c_{2}}{\sigma_{x}^{2}+\sigma_{y}^{2}+c_{2}} \end{array}$$

(12) $$\begin{array}{cc} s(x,y) & =\frac{\sigma_{xy}+c_{3}}{\sigma_{x}\sigma_{y}+c_{3}} \end{array}$$

where $\mu_{x}$ is the mean of *x*, $\mu_{y}$ is the mean of *y*, $\delta_{x}^{2}$ is the variance of *x*, $\delta_{y}^{2}$ is the variance of *y*, $\sigma_{xy}$ is the covariance of *x* and *y*, $c_{1}={\left(k_{1}L\right)}^{2},c_{2}={\left(k_{2}L\right)}^{2}$ are two constants needed in order to avoid dividing by 0, *L* is the range of pixels, and $k_{1}$ = 0.01 and $k_{2}$ = 0.02 are the default values.

Then, we have

(13) $$\mathrm{SSIM}\left(x,y\right)=\left[l{\left(x,y\right)}^{\alpha }\cdot c{\left(x,y\right)}^{\beta }\cdot s{\left(x,y\right)}^{\gamma }\right]$$

Set α,β,γ as 1. Then there is:

(14) $$\mathrm{SSIM}\left(x,y\right)=\frac{\left(2\mu_{x}\mu_{y}+c_{1}\right)\left(2\sigma_{xy}+c_{2}\right)}{\left(\mu_{x}^{2}+\mu_{y}^{2}+c_{1}\right)\left(\sigma_{x}^{2}+\sigma_{y}^{2}+c_{2}\right)}$$

**Figure 4:**
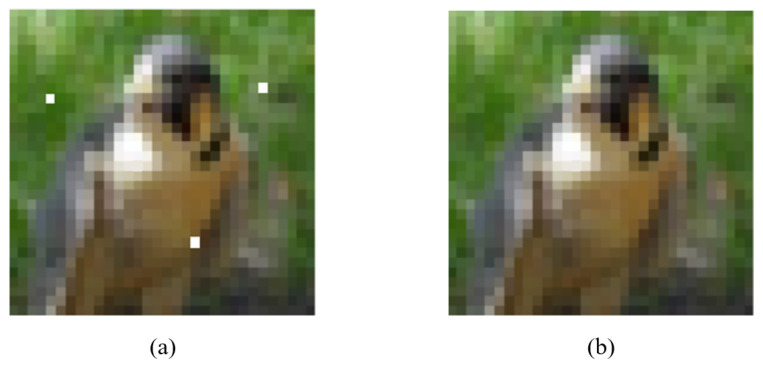
Adversarial sample and adversarial stego-image with embedded information: (**a**) BP–DE processed adversarial sample; (**b**) ADH processed adversarial stego-image.

**Figure 5:**
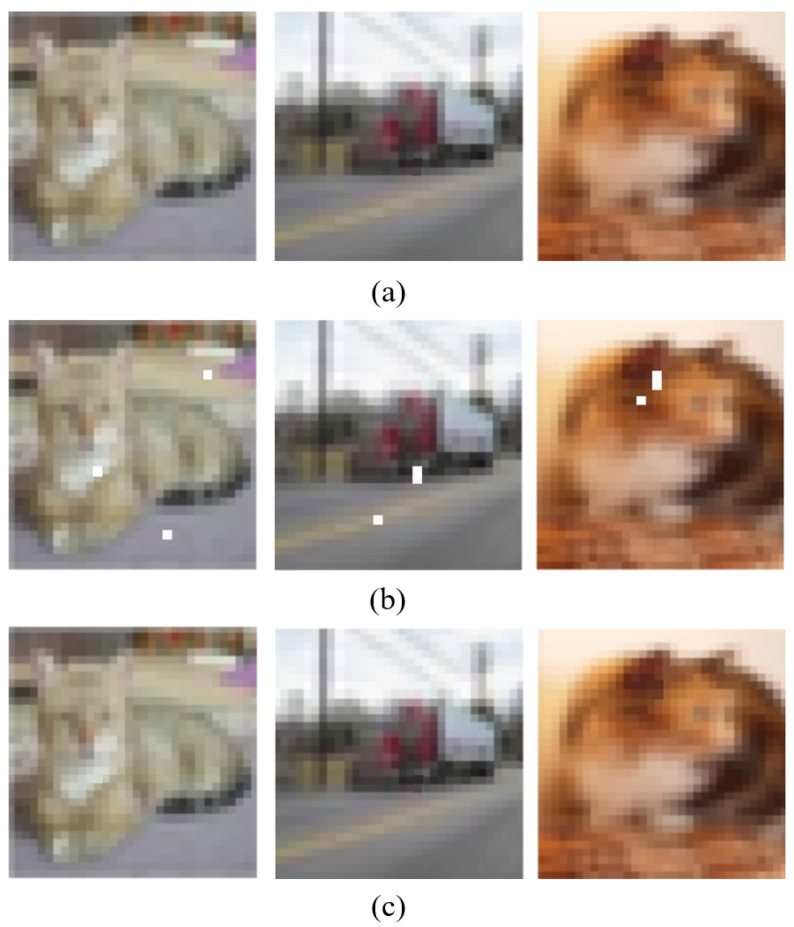
Comparison of the original images, adversarial images, and adversarial stego-images: (**a**) the original images; (**b**) the BP–DE processed adversarial images; (**c**) the ADH processed adversarial stego-images.

Each calculation takes a window of N×N from the image, continues sliding the window for calculation, and finally takes the average value as the global SSIM. The ADH image’s steganography evaluation is simply an image quality assessment of the adversarial stego-image. As shown in Figure 4, we used an image from the CIFAR-10 bird dataset as a demonstration. The adversarial sample of Figure 4a was created by using the BP–DE algorithm to locate three discrete “sensitive pixels” on the decision boundary. The adversarial stego-image is the image of Figure 4b, which contains information embedded in the adversarial sample using the LSB technique. The encoded data consists of the nine bits and can be fully extracted. The embedded information of these two images is “CIFARBIRD”, and the complete embedded information can be obtained after decoding. It is worth noting that dividing the “sensitive pixels” into separate individuals improves the image quality. This result demonstrates that ADH-generated images may maintain the secret data hidden while considerably improving the image quality of the confrontation adversarial sample. As illustrated in Figure 5, the cat in the first column is misclassified as a frog, the truck in the second column is misclassified as a deer, and the cat in the third column is misclassified as a dog. We can clearly see that while embedding information, the images are aggressive.

Next, we perform a subjective evaluation of the secret confrontation images. The images in Figure 5a,c can be seen with the naked eye, leading to the conclusion that the adversarial stego-image is visually quite similar to the original image. We employed PSNR to objectively analyze the adversarial stego-image and obtain a quantifiable number for the image quality evaluation based on error sensitivity in order to avoid this false visual perception result being influenced by the numerous subjective elements. The PSNR of this adversarial stego-image is 40.23112 dB, while the average PSNR of 500 adversarial stego-images is 41 dB, meaning that the image quality is superb. The average PSNR of 500 hidden adversarial images is 0.96, and the SSIM is 0.9562. The adversarial stego-image was found to be visually extremely similar to the original image.

**Figure 6:**
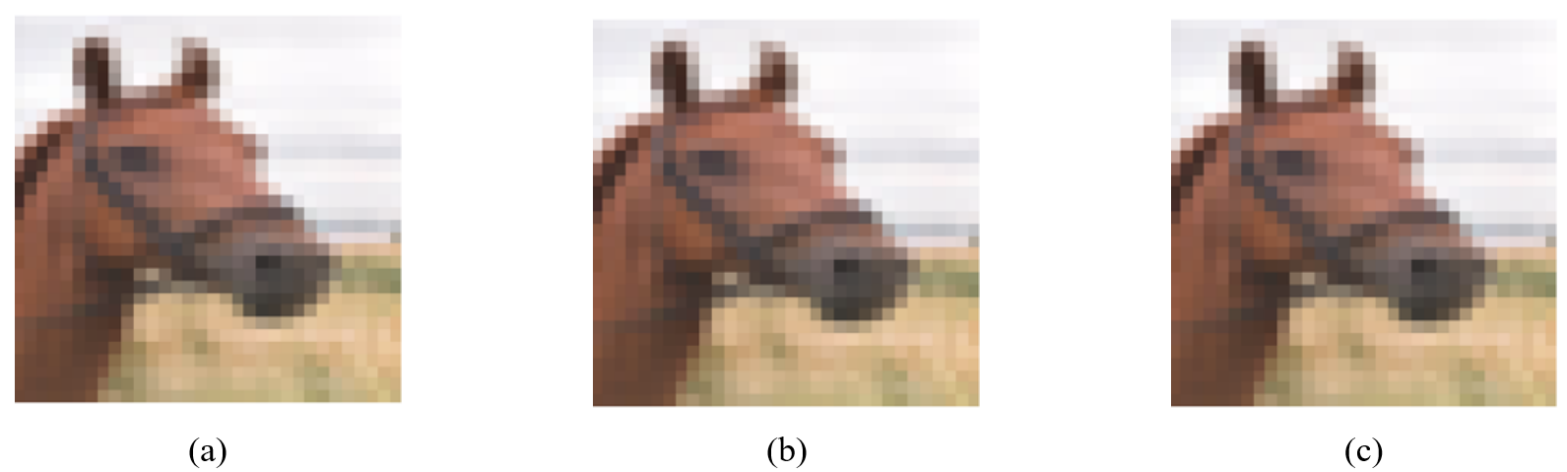
Image quality comparison of multiple images processed by ADH: (**a**) the image modified by ADH for one pixel has a PSNR value of 41.37; (**b**) the image modified by ADH for three pixels has a PSNR value of 41.03; (**c**) the image modified by ADH for five pixels has a PSNR value of 40.85.

Our proposed ADH method greatly reduces the impact of embedding capacity enhancement on image quality. As shown in Figure 6, although the increase in embedding capacity decreases the PSNR value, the magnitude is very small, and is almost negligible when reflected visually. This is a characteristic of the LSB algorithm itself, as it is the first five bits of the color channel of each pixel, the MSB, which seriously affects the quality of the image. However, the LSB algorithm only modifies the last three bits when hiding the information, leaving the first five bits unchanged. Therefore, as more pixels are changed, the quality of the image is not greatly affected.

### 3.2. Aggressiveness Test

We first verified the restriction modification unit and the number of disturbed pixels, then used the BP–DE algorithm to explore the decision boundary in the search space, locate the “sensitive pixel” along the decision boundary, and disturb the pixel to see whether the attack effect persisted.

**Figure 7:**
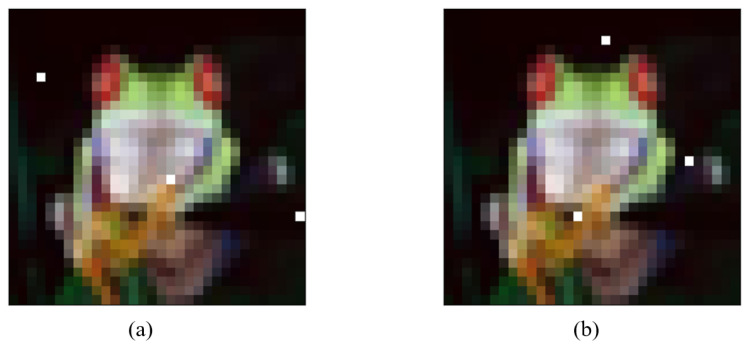
Different adversarial samples of the same original image: (**a**) The true label is frog, and the predicted label is cat; (**b**) The true label is frog, and the predicted label is dog.

As shown in Figure 7, after limiting the modification unit and constraining the amount of modified pixels, it is possible to locate the decision boundary in the search space using BP–DE. The ordinary sample can become adversarial by randomly selecting discontinuous “sensitive pixels” on the decision border and changing the color of these pixels to make them perturbations. It is worth noting that the CNN network can misclassify both of the Figure 7 adversarial samples, although their ”sensitive pixel” positions are not the same. Furthermore, as shown in Figure 7a,b, when different “sensitive pixels” are selected the discriminator performs different classes of misclassification. This demonstrates the BP–DE algorithm’s randomness and shows that the BP–DE algorithm actually scans the search space for decision boundaries and finds discontinuous ”sensitive pixels.”

**Figure 8:**
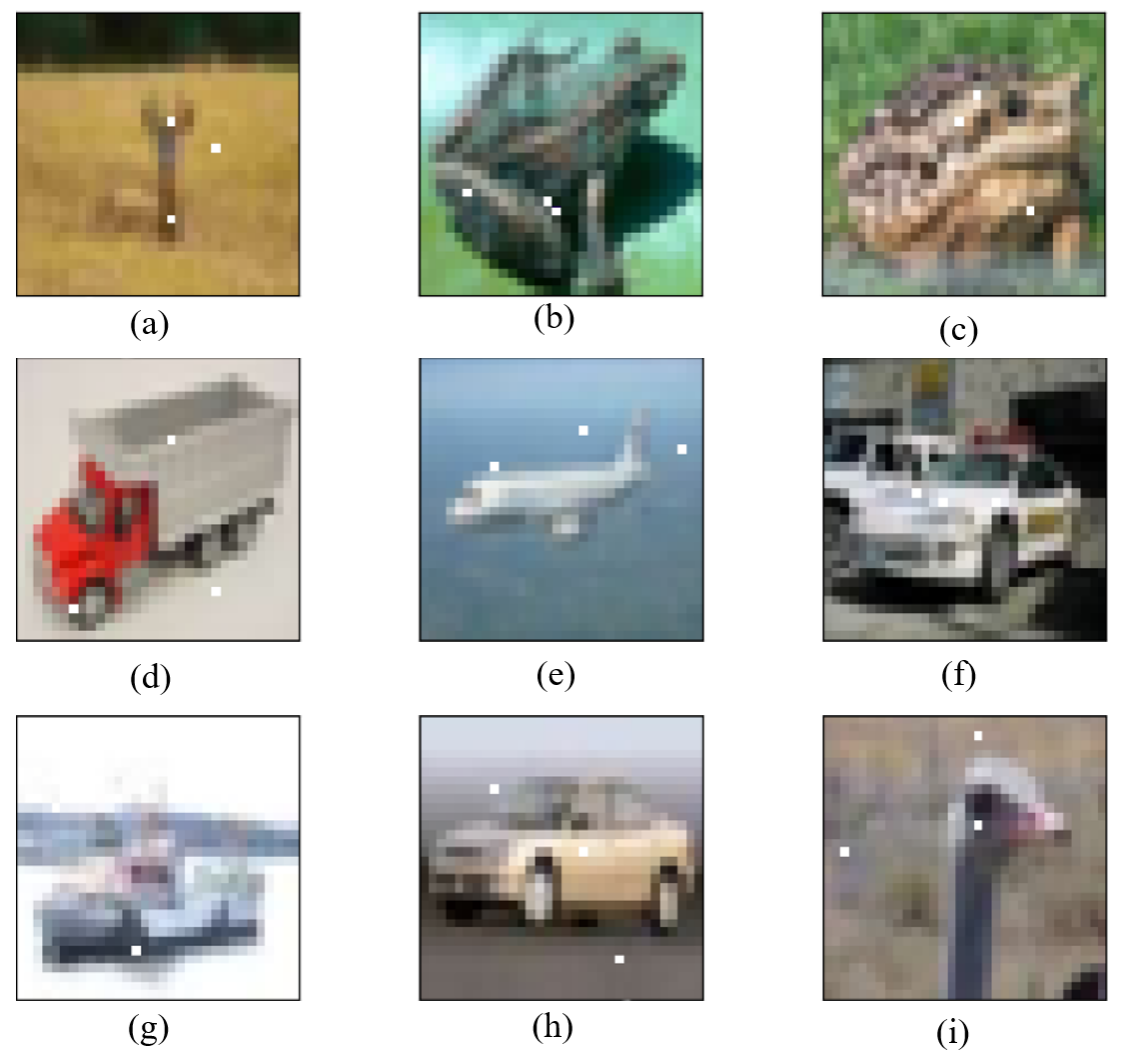
Adversarial samples generated by using BP-DE: (**a**) In Lenet, deer is predicted as dog; (**b**) In Lenet, frog is predicted as automobile; (**c**) In Lenet, frog is predicted as deer; (**d**) In Net in Net, truck is predicted as airplane; (**e**) In Net in Net, airplane is predicted as ship; (**f**) In Resnet, automobile is predicted as truck; (**g**) In Lenet, ship is predicted as automobile; (**h**) In Resnet, automobile is predicted as truck; (**i**) In Lenet, bird is predicted as dog.

As shown in Figure 8, the BP–DE algorithm, as described in Section 2, makes the ADH flexible and suitable to a wide range of CNNs. In Lenet, as shown in Figure 8a–c,g,i, the true labels are respectively deer, frog, frog, ship, and bird. However, the predicted labels are respectively dog, automobile, deer, automobile, and dog. In Resnet, as shown in Figure 8f,h, the true labels are automobile and airplane, respectively, while the predicted labels are truck and truck, respectively. In Net in Net, as shown in Figure 8d,e, the true labels are truck and airplane, respectively, while the predicted labels are airplane and ship, respectively. As a result, it can be trained on different networks to generate adversarial samples, and the adversarial samples thus generated can successfully fool the CNNs. This can explain why we were able to cut down the number of modification units. We only modified three pixels and successfully affected a large number of images, as the CNN is sufficiently sensitive to tiny perturbations.

The aggressiveness of the adversarial stego-images generated by ADH are considered below, along with two metrics are introduced here in order to comment on the aggressiveness of the hidden adversarial samples.

- Accuracy: the percentage of misclassification of the discriminator in all samples, or the number of samples in the adversarial sample as a proportion of the total number of samples. This metric illustrates the ability of the adversarial stego-images to generate adversarial samples.
- Successful attack rate: the attacks used in this paper are non-targeted attacks, which are defined as the percentage of misclassified attacks. As long as the discriminator classifies the adversarial stego-image into the wrong category, it is considered a successful attack.

**Figure 9:**
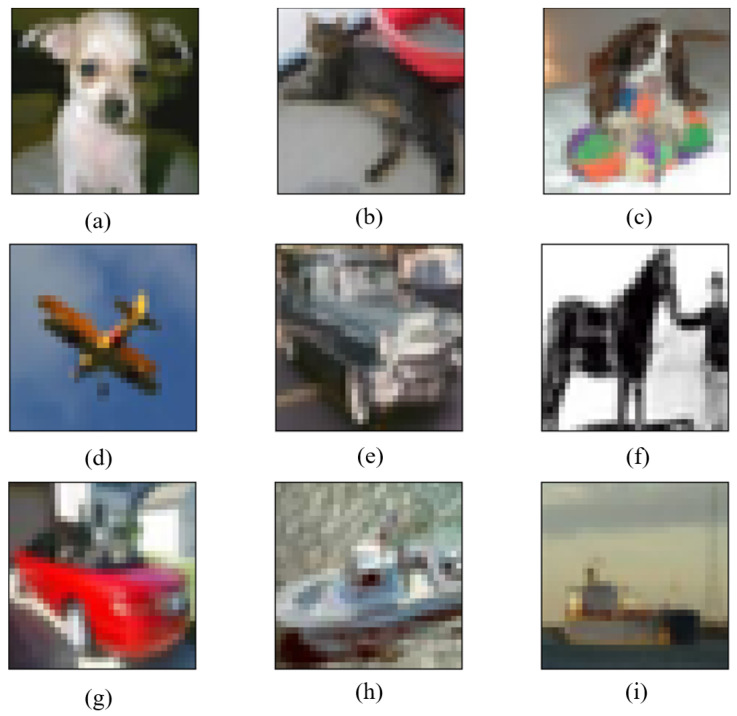
Adversarial stego-image generated by ADH: (**a**) in Lenet, dog is predicted as cat; (**b**) in Resnet, cat is predicted as automobile; (**c**) in Resnet, dog is predicted as horse; (**d**) in Resnet, airplane is predicted as automobile; (**e**) in Net in Net, automobile is predicted as truck; (**f**) in Lenet, horse is predicted as automobile; (**g**) in Net in Net, automobile is predicted as airplane; (**h**) in Lenet, ship is predicted as automobile; (**i**) in Net in Net, ship is predicted as truck.

The data and images reveal that the adversarial stego-images are aggressive. Figure 9 shows that ADH can be applied in various CNNs. In Lenet, as shown in Figure 9a,f,h, the true labels are respectively dog, horse, and ship, while the respective predicted labels are cat, automobile, and automobile. In Resnet, as shown in Figure 9b–d, the true labels are cat, dog, and airplane, respectively, while the predicted labels are automobile, horse, and automobile, respectively. In Net in Net, as shown in Figure 9e,g,i, the true labels are automobile, automobile, and ship, respectively, while the predicted labels are truck, airplane, and truck, respectively. Table 1 shows that adversarial stego-images are highly able generate adversarial samples. After the adversarial stego-image successfully interferes with the CNNs to misclassify it, the CNNs’ discriminator will be very confident that it is the wrong category. Because the adversarial stego-images are generated by modifying the ”sensitive pixels” on the decision boundary, we can conclude that the decision border has a significant impact on the CNN’s judgment.

At the same time, we found that the adversarial stego-images are slightly less aggressive than the normal adversarial samples. Because the adversarial sample’s concealment and aggression remain contradictory, if the adversarial sample’s visual quality is to be increased it must come at the expense of decreased aggressiveness. However, in the adversarial sample that cannot exist, ADH moderates the relationship between concealment and aggressiveness and embeds secret data. These results are sufficient to demonstrate that ADH represents a massive breakthrough in the combination of data hiding and GAN techniques.

### 3.3. Metrics Comparison

Many GAN-based data hiding algorithms, as shown in the first section of this paper, cannot guarantee security and extraction accuracy at the same time, among them the IHGAN algorithm and the Secure-IHGAN algorithm  [28]. The IHGAN algorithm, which adaptively hides data in a carrier image and effectively resists steganalyzer detection. The Secure-IHGAN algorithm encrypts secret information by adding a secret key to the previous one, while the adversarial stego-image proposed in this paper, ADH, generates the carrier image without visual distortion. Security represents the ability to resist steganalysis model detection; however, it implies the authenticity and low distortion rate of the carrier image as well. Even if the carrier picture generated by the steganography models is undetectable by the steganalysis models, it is unacceptable that the image is noticeably abnormal and unnatural to the naked eye. In this paper, we conduct a comparison in order to evaluate the quality of the generated image using three methods.

**Figure 10:**
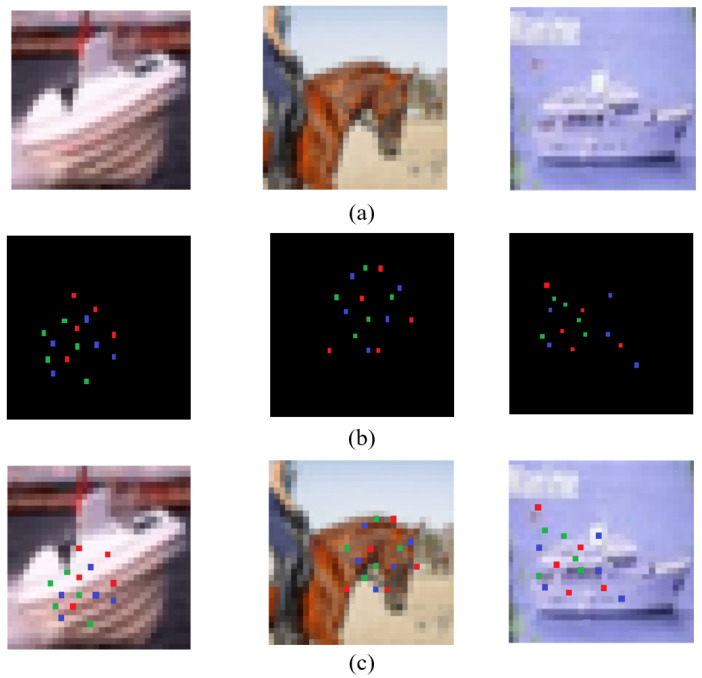
Traditional multi-pixel adversarial perturbation: (**a**) the original images; (**b**) the visible perturbations to be added to the images; (**c**) the images with added perturbations.

The image quality with the proposed ADH approach and the presently available techniques are compared in Figure 10, using the traditional multi-pixel perturbation to generate adversarial samples. As seen in Figure 10c, traditional perturbation is incapable of serving the goal of steganography, let alone hiding information. This substantially degrades image quality and makes it easier for unethical components to create a perturbation model by repeatedly learning diverse adversarial samples. As a result, the image’s security is severely compromised.

As shown in Section 3, another advantage of the adversarial stego-images is that the encoded information can be 100% extracted by decoding, which means that the extraction accuracy is high. The precision of secret information extraction is critical to the practicality of the steganographic model. In order to improve the robustness of the model and boost the correctness of the information extracted by the decoder, the Robust-IHGAN method [28] adds a noise layer to IHGAN to simulate picture distortion which may be faced during the transmission of the secret image. Table 2 shows the comparison outcomes, which compare the extraction accuracy of these three methods. The IHGAN model has a 50% correct decoding rate before adding the noise layer; however, the Robust-IHGAN model has a high decoding accuracy of over 80%. Obviously, these algorithms cannot compete with the decoding accuracy of ADH. In most cases, the extraction accuracy of the proposed ADH method exceeds the level of the prior state-of-the-art, showing that the proposed ADH method can ensure that secret information is transmitted securely and completely.

The validation experiments above aloow us to conclude that the performance of our proposed ADH algorithm in terms of image quality, and extraction accuracy is far superior to the adversarial stego-images based on machine learning proposed by [28]. To the best of our knowledge, the idea of generating adversarial samples via information hiding by combining the two techniques has not previously been proposed in the field. At present, there are two general ways of generating adversarial samples: (1) adding noise; and (2) using watermarks which are visible. Because of the principle of generating adversarial samples, this technique has an extra feature of information hiding when compared to the first method. Compared to the second method, as stated in the Introduction section and the discussion of the explicit watermarks of [21,22], although it achieves information embedding, it cannot protect the picture quality as effectively as an implicit watermark, and can even be extracted, endangering security.

## 4. Conclusions

This paper combines data hiding and adversarial sample generation, and proposes the first data hiding method, ADH, which has both a concealment and an adversarial function. ADH can secretly embed information and transform ordinary samples into adversarial samples without compromising image quality. It is a promising technique that ensures the invisibility of the embedded information, the adversarial nature of the sample, and the high quality of the image, with many flexible application scenarios. Our analysis and experiments confirm the effectiveness of this approach. In the future, the ADH algorithm might be used in the fields of facial recognition and copyright protection to contribute to the safe transmission of information and the prevention of personal information leakage.

## Figures and Tables

**Table 1:** Attack success rate and accuracy of adversarial stego-images generated by ADH in three types of CNNs.

CNN	Accuracy	Attack Success Rate
LeNet	74.88%	3.8%
Net in Net	80.77%	3.2%
ResNet	51.33%	2.0%

**Table 2:** Decoding accuracy comparison of ADH with IHGAN  and Robust-IHGAN.

Algorithm	Decoding Accuracy
IHGAN [28]	50%
Robust-IHGAN [28]	90∼98%
ADH	100%

## Data Availability

The datasets generated during the current study are available from the corresponding author on reasonable request.

## Appendix A. Algorithm

**Algorithm A1** Adversarial Data Hiding Algorithm.
Require: img, network_model, n, maxiter, popsize, img_class, img_confidence, img_rgb Ensure: $img_{ADH}$ 1: Input the secret message 2: Divide the secret message into n*3 pieces 3: Set the img and size of popsize, maxiter, network_model, n 4: Randomly initialize location coordinates of the parent population according to (5) 5: Define population vector $X_{s}=\;(x_{1},y_{1},x_{2},y_{2},\cdots ,x_{n},$ $y_{n})$ 6: Set upper and lower bounds of variables x,y 7: Set rgb values of the all populations are (255,255,255) 8: Read img_class, img_confidence, img_rgb 9: **for**g=1,⋯,maxiter**do** \\*g* is the generation number 10: Add the newly generated scrambled pixels to the image and observe the confidence 11: **for** i=1,⋯,popsize **do** 12: The generation of mutant offspring according to (6) 13: **end for** 14: New populations are selected according to network_models’ confidence and class 15: Update $X_{s}$ 16: **end for** 17: Select the optimal solution of this generation as $X_{s}\left(g+1\right)$ 18: Get the value of $x_{1},y_{1},x_{2},y_{2},\cdots ,$$x_{n},y_{n}$ based on $X_{s}\left(g+1\right)$ 19: **for** i=1,⋯,n **do** 20: Read the original rgb of $\left(x_{i},y_{i}\right)$ from img_rgb 21: r, g, b = pixel[1], pixel[2], pixel[3] 22: r, g, b = bin(r), bin(g), bin(b) \\Change rgb as 8-bit binary numbers 23: **for** j=1,⋯,n **do** 24: r[j] ← r[0:5] + secret message1[j] 25: g[j] ← g[0:5] + secret message2[j] 26: b[j] ← b[0:5] + secret message3[j] 27: **end for** 28: Generate $img_{ADH}$ 29: **end for** 30: **Final:** 31: **return** $img_{ADH}$
